# Correlation Seminar during Basic Medical Science: Our Experience

**DOI:** 10.31729/jnma.8445

**Published:** 2024-02-29

**Authors:** Dhirendra Rokaya, Neetika Paudel

**Affiliations:** 1 KIST Medical College and Teaching Hospital, Imadol, Lalitpur, Nepal; 2 Maharajgunj Medical Campus, Maharajgunj, Kathmandu, Nepal

**Keywords:** *learning*, *lectures*, *medical school*, *medical students*

## Abstract

Correlation seminars are used in teaching curricula as a strategy to encourage students to build a solid foundation in basic science at the beginning of the learning process. Establishing a link between basic science and medical practice is the main objective of the correlation seminar which helps students relate topics from basic science with a medical application or disease. Correlation seminars are designated in such a way that students can apply their basic science knowledge acquired from lectures and practical classes in clinical patient care. Course-centred problem-solving emphasises the identification and resolution of clinical issues to enhance clinical reasoning abilities. Through correlation seminars, students are allowed to engage in critical thinking and apply their knowledge of basic science to clinical settings.

## INTRODUCTION

A correlation seminar is an active instructional method that has been introduced in our curriculum, enabling students to actively engage in their education through discussion, debate, querying, and cooperation with teachers and peers.^1^ The seminar teaching approach is a successful way to raise student collaboration, knowledge and skill scores, active learning capacity, classroom environment, and teacher-student engagement.^2^ Correlation seminar helps students become more interested in the subject matter, improve their clinical reasoning skills, and also their long-term retention of basic science knowledge.^3^ The first two years of basic science are the pillars for developing a professional career and the correlation seminar made us actively participate in learning basic medical science.^4^ In this article, we have shared our learning experiences from correlation seminars and how we used them for our learning and growth.

## OUR EXPERIENCE

The university we are studying at currently includes correlation seminars in the curriculum to promote integrated active learning among students that can be used in clinical studies. Incorporation of clinical topics in correlation seminars enables the teaching of basic science subjects and aids in the development and practice of strong clinical abilities in students.^1^ It is mostly conducted at the end of each organ system, and the faculty members decide the topics.^5^ For example, while having a correlation seminar at the end of the cardiovascular system, we were given the main topic of myocardial infarction, and along with that, we were also given two learning objectives from each of the respective departments of basic science. Like in anatomy, our learning objectives were about the conduction system of the heart and the blood supply of the heart. Students make presentations under different sub-topics of one main topic to meet the objective of the topic assigned, which is reviewed by the faculties. The presentation skills and understanding of the concept were evaluated, and areas for improvement were marked. It also helped in personality development by improving our interpersonal skills while communicating with our teachers and colleagues. A correlation seminar is designed to relate learning from different subjects to the relevant case scenario.

Students were divided into different groups, each with 10 students, and each of them would get one sub-topic. The sub-topics would be from different subjects of basic science, and students would be given a deadline to prepare the slides. Then the slides are reviewed by the faculty members, and students edit them as per their suggestions. While making slides, students have to correlate the knowledge of one subject with another to solve the given objectives.

Slides are prepared according to the guidelines provided by the teacher, with an emphasis on making the slides more figurative by adding graphs, charts, figures, or as per the requirement given. Standard textbooks, reference books, and notes from our classroom lectures were used and presented within the given time frame. Faculty members representing each department were present during the presentation, and they would evaluate us based on the content, way of delivery, and answers to the questions followed by our presentation. They would even give us constructive feedback about how to improve the errors that were made throughout the sessions. The feedback was valuable for us to make the presentation better in the following sessions. The evaluation would also be considered during our formative assessment.

## OUR LEARNINGS

Correlation seminar drives students to engage in critical thinking and apply knowledge of basic science to the clinical setting.^6^ The correlation seminar helped us to improve our understanding of the content of basic sciences along with that it made our learning easier and longer-lasting. Students interrelate the knowledge of one subject to another, through which they were able to make our study more effective and practical. Students learn about the features of Microsoft PowerPoint during slide preparation, including slide design, format, style, etc. Even though it was difficult to work in groups, we learned about the various aspects of group dynamics. Following the correlation seminar, students encountered the process of cultivating the habit of asking and linking things while learning. They improved the presentation's relevance and informational value by using the feedback provided by our teachers. At first, we were quite reluctant to talk to the faculties, but as time went on, we improved the interpersonal and communication abilities, which helped us with our own skill development. Students were able to get over their stage fright and anxiousness during their presentation in front of the mass, and things improved over time. Through the question and answer session at the end of the presentation, every student got to evaluate their knowledge of the particular subject matter. That further encouraged us to seek more content and explore it. Correlation seminars are conducted based on a designed syllabus of basic science which was also helpful according to the exam point of view.

## WAY FORWARD

Correlation seminars are important in the basic sciences for promoting self-learning and active participation in learning among medical students. The correlation seminar can work as a revision of important topics as it is conducted after teaching a particular human body system per curriculum. When a single medical topic is related to six different integrated subjects of basic science, it makes learning easier and smoother, which helps in retaining the knowledge for a long time. The correlation seminar also interrelates basic and clinical science while integrating knowledge from all the subjects. For active learning, building organisational skills, and learning teamwork among medical school students, this learning modality can be practised more in medical institutions.
